# Intervertebral Disc Magnetic Resonance Spectroscopy Changes After Spinal Manipulative Therapy for Lumbar Discogenic Pain

**DOI:** 10.7759/cureus.72225

**Published:** 2024-10-23

**Authors:** Jessica F Billham, Dingbo Shi, Erika Evans Roland, Matthew F Gornet, Kelly K Brinkman, Francine W Schranck, James M Cox, Norman W Kettner

**Affiliations:** 1 Department of Radiology, Logan University, Chesterfield, USA; 2 Department of Clinical Chiropractic, Montgomery Health Center, Logan University, Chesterfield, USA; 3 Department of Orthopedics, The Orthopedic Center of St Louis, St Louis, USA; 4 Department of Chiropractic, College of Chiropractic, Logan University, Chesterfield, USA; 5 Department of Research, SPIRITT Research, St Louis, USA; 6 Department of Radiology and Clinical Chiropractic, Cox Chiropractic Medicine, Fort Wayne, USA

**Keywords:** chiropractic, cox flexion-distraction, low back pain, magnetic resonance spectroscopy, spinal manipulative therapy

## Abstract

This study investigates the use of magnetic resonance spectroscopy (MRS) to identify the intervertebral disc (IVD) as a pain generator, explore the pathophysiology of the biochemical and structural components of discogenic low back pain (DLBP), and present potential evidence of physiological responses to spinal manipulation therapy (SMT). A 29-year-old male presented with uncomplicated low back pain (LBP). The non-specific presentation and clinical examination findings were consistent with an initial working diagnosis of non-specific LBP with the clinician suspecting IVD as a likely pain generator. Conventional magnetic resonance imaging showed findings of IVD degeneration including Modic type I changes consistent with a diagnosis of DLBP. MRS was utilized for structural and biochemical analysis of the IVDs. Altered spectral features confirmed a DLBP diagnosis. The patient underwent 16 Cox flexion-distraction SMT treatments at a chiropractic teaching clinic in Chesterfield, Missouri. A follow-up MRS was performed to compare and evaluate post-treatment results.

We report the utilization of MRS to quantify the structural integrity and biochemical pain profile of the IVD in a conservatively managed chronic DLBP patient who was unresponsive to previous steroid injections. Comparison between MRS revealed improved IVD spectral features including decreased biochemical pain markers and increased glycoprotein biosynthesis. This implies that the SMT management of chronic DLBP may improve IVD structural integrity and alter pain biochemistry.

## Introduction

Low back pain (LBP) is a common health problem and cause of disability affecting upwards of 80% of adults in their lifetime [1-4]. In the United States, LBP healthcare costs have increased substantially in the past decade, making this health problem a burden on both the individual and society [2,3]. Pain is multifaceted including physiologic and psychosocial factors that should be considered when evaluating all LBP cases [5-7].

Currently, 90% of LBP is classified as non-specific (NSLBP) and lacks a clear pathoanatomical etiology, or anatomical target [8]. Francio et al. stated how spine care should “aim to pursue the right treatment for the right patient at the right time, and (be) directed to the correct anatomical target” [9]. Specific LBP, however, has a clear pathoanatomic source, either spinal or non-spinal in origin [10,11]. Often discogenic low back pain (DLBP) is initially classified as NSLBP due to vague presentations and inconclusive diagnostic test results; however, positive imaging findings can lead to the diagnosis [12].

Qualitative imaging dominates attempts to specify a pathoanatomic source in NSLBP; however, imaging findings correlate poorly (low specificity) with clinical presentations [13]. Qualitative imaging may show intervertebral disc (IVD) degeneration, such as reduced IVD height and endplate sclerosis on lumbar radiography and reduced IVD fluid signal intensity on conventional magnetic resonance imaging (MRI) [14-16]. Pfirrmann grading is a common method to evaluate IVD degeneration on MRI but is insensitive to early biochemical alterations. Instead, it centers on the IVD fluid signal intensity, and distinctions between separate IVD structures and IVD height. It is a five-class system with 1 representing normal and 5 representing severe degeneration [17,18]. Provocative discography is another qualitative IVD analysis performed by injecting contrast into the suspected IVD to highlight morphologic changes under fluoroscopy [5,15].

The IVD is considered the pathoanatomic source of LBP when there is LBP with conventional MRI evidence of IVD degeneration, also termed “active discopathy” [19,20]. When the IVD undergoes degeneration, many structural and biochemical changes occur that may trigger pain. Degenerative IVD findings on MRI include Modic type I bone marrow edema, annular fissures or herniation of the nucleus pulposus (NP) due to loss of IVD structural integrity, and high-intensity zones [7,14,21-24]. However, these findings may be visualized in asymptomatic patients [14-16].

To support qualitative MRI findings, quantitative imaging such as magnetic resonance spectroscopy (MRS) can quantify the structural and biochemical changes that occur with IVD degeneration [5,17,25,26]. Metabolites like proteoglycans and lactate serve as biomarkers to identify specific IVDs as the pathoanatomic source of pain when quantified using novel NOCISCAN technology (Broomfield, CO: Aclarion Inc.) [21,24].

Patients with DLBP should be carefully paired with appropriate treatment options. This may include surgery, analgesic prescription, and/or conservative care. Non-surgical, non-pharmacologic treatments are the most common interventions including patient education, spinal manipulative therapy (SMT), and exercise [27]. In fact, for some patients, these conservative options may be as effective as surgery and opioids in reducing pain but are more cost-effective with a lower risk of complications [28,29].

We describe a case of NSLBP with qualitative lumbar IVD degeneration suggesting a DLBP diagnosis. MRS analysis of the lumbar IVDs quantified structural and biochemical changes indicating the IVD as the pathoanatomic source of pain. The IVD structural integrity and degenerative pain biochemistry after a course of Cox flexion-distraction SMT were markedly improved. This is the first report of MRS utilized to quantify IVD structural integrity and biochemical changes in baseline and post-SMT for a case of DLBP. This aimed to show the utility of MRS to identify the IVD as a pathoanatomic source of pain in LBP patients, review the current understanding of the structural and biochemical components of DLBP, and demonstrate how conservative management of DLBP with Cox flexion-distraction SMT may provide benefit.

This case was previously presented as a meeting abstract at the Association of Chiropractic Colleges Educational Conference and Research Agenda Conference 2023: Leadership in Education on March 25, 2023. This article was previously posted as a preprint on the Research Square server between April 04, 2023, and February 06, 2024.

## Case presentation

A 29-year-old male office worker and military reservist presented to a chiropractic teaching clinic with an acute flare of LBP of two days duration. His goals were to reduce pain, return to weight-lifting and golfing, and improve function for a long-term active lifestyle. Maintaining an adequate level of fitness to perform necessary military duties was important to him.

Prior episodes of similar pain occurred over the past several years that the patient attributed to bounding out of helicopters while on active military duty. The patient sought care at the emergency department for a comparable episode approximately one year prior and received an epidural steroid injection and pain medications which provided minimal relief. Subsequent physical therapy provided no perceived benefit. The last episode of pain was six months prior and was self-managed with rest, ice, and over-the-counter analgesics until resolution over the course of three months.

The presenting pain was rated 4 on the Numeric Pain Rating Scale (NPRS) and localized to the lower lumbar region without radiation into the extremities. It was described as a constant deep ache that progressed to sharp pain with lumbar flexion. Standing and stretching provided some relief. The Oswestry Disability Index (ODI) was 34%, representing moderate disability. The patient denied experiencing night pain, night sweats, unexplained weight loss, or uncontrolled bowel/bladder function. Psychosocial contributors included fear-avoidance of tasks related to military physical fitness requirements, such as deadlifting and running.

The patient presented without antalgia, although needed support when transitioning postures for fear of increased pain. Clinical examination of lower extremity dermatomes, myotomes, and deep tendon reflexes revealed normal neurological function. The lumbar paraspinal musculature was taut and tender bilaterally. Active lumbar ranges of motion in flexion, extension, and lateral bending were full but reproduced pain. The patient’s pain was provoked by Yeoman and Kemp's orthopedic tests bilaterally. Milgram and Dejerine triad were negative.

**Figure 1:**
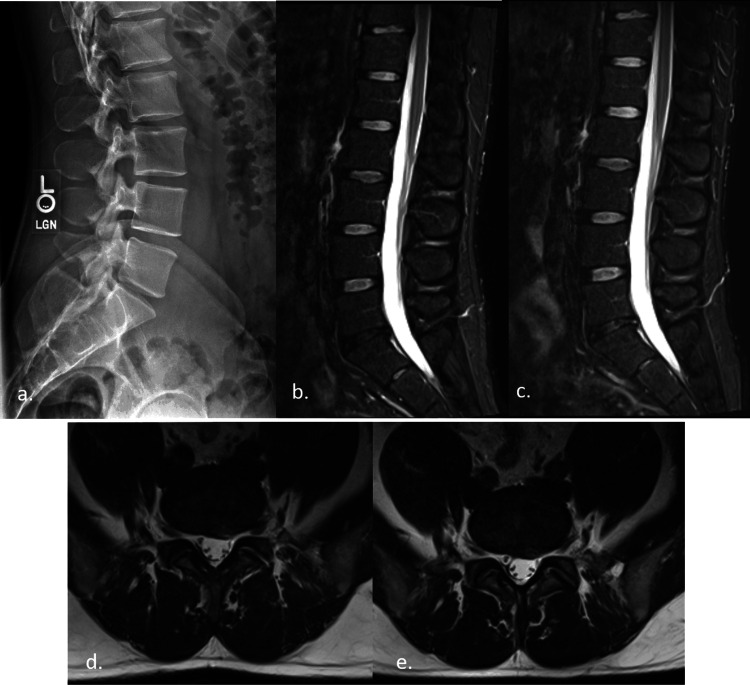
Baseline imaging shows changes in the L5/S1 IVD space and disc conditions, with no significant post-treatment improvement. Baseline lateral lumbar radiographs showed a minimal decrease in the L5/S1 IVD space (a). Baseline sagittal STIR sequence demonstrates Pfirrmann grade 3 at L5/S1 (b) without qualifiable interval change in the post-treatment image (c). Baseline axial T2-weighted sequence shows a disc protrusion with high-intensity zone mildly effacing the thecal sac and left S1 nerve root (d) without qualifiable interval change post-treatment (e). STIR: short tau inversion recovery; IVD: intervertebral disc

Based on the non-specific clinical presentation and absence of red flags, the patient was diagnosed with NSLBP with suspicion of discogenic etiology and identified as a good candidate for SMT. Because fear-avoidance behaviors were present, reassurance and education about the benign and self-resolving nature of LBP were emphasized throughout treatment. Even after patient education was provided, the patient remained recalcitrant to increasing physical activities. After three sessions of Cox flexion-distraction SMT over three weeks without improvement, lumbar radiography was acquired (Figure 1, panel a). A lumbar MRI was obtained six weeks after initiation of care for qualitative evaluation of the IVDs (Figure 1, panels b, d).

**Figure 2:**
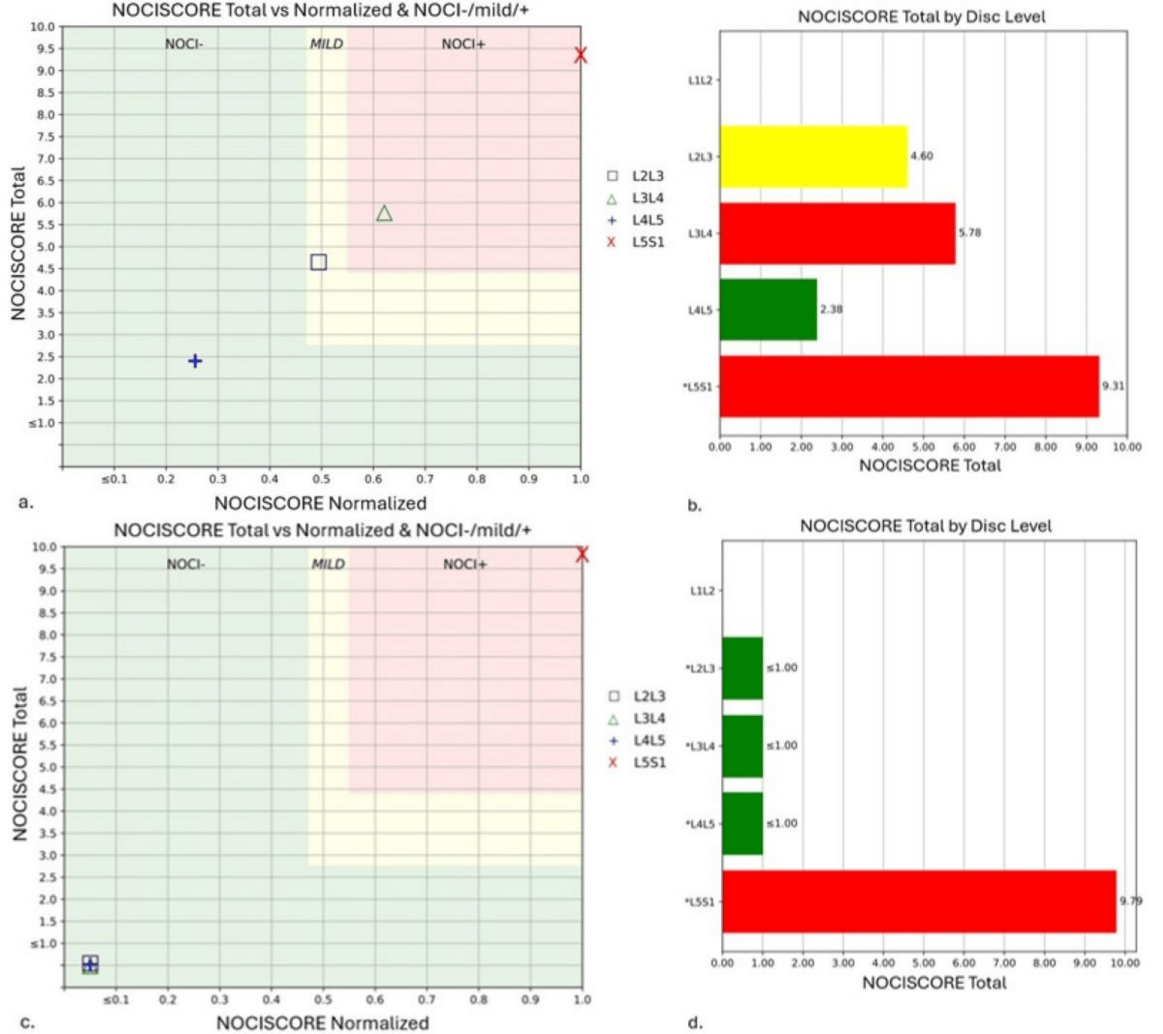
Baseline NOCISCORE analysis shows L3/4 and L5/S1 levels correlated with pain; and, post-treatment scores indicate improved pain for L2/3 to L4/5, but a slight rise at L5/S1. The NOCISCORE is a ratio of proteoglycans to lactate that has been correlated with provocative discography results to distinguish which IVDs are painful. Correlation to positive provocative discography is graphed in the red zone while negative correlation is in the green zone. Baseline NOCISCORE (a, b) shows the L3/4 and L5/S1 IVDs in the red zone which indicates a correlation to a positive provocative discography and therefore likely a pathoanatomic source of pain. The post-treatment NOCISCORE (c, d) demonstrates a predicted significant drop in the pain scores of L2/3 through L4/5, but a mild increase at L5/S1, likely due to a patient's exacerbation. IVD: intervertebral disc

**Figure 3:**
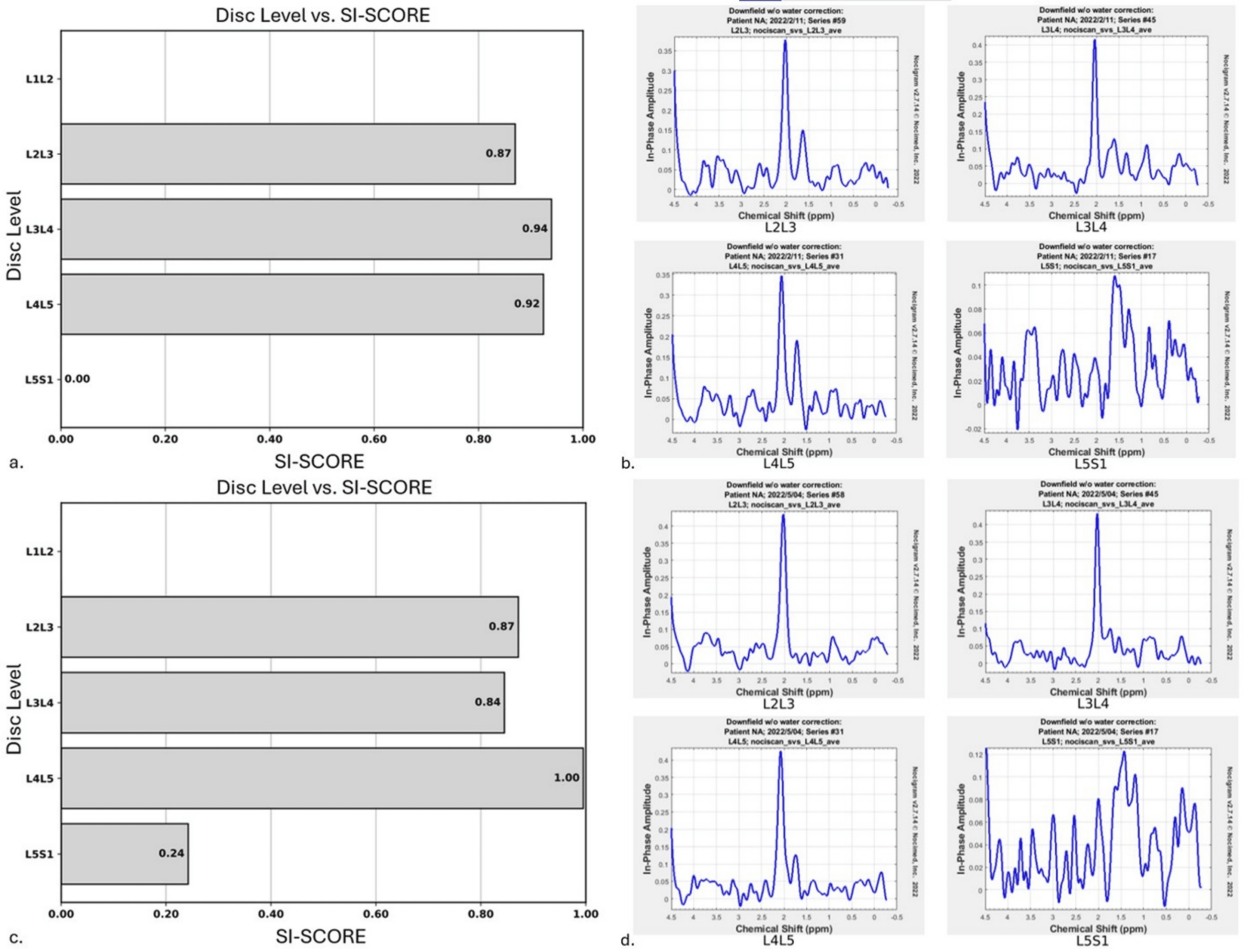
Improved proteoglycan levels at L5/S1 post-treatment, with spectral graphs highlighting significant recovery. The SI score represents structural integrity and the quantity of proteoglycans present. In baseline analysis, the L5/S1 disc level of proteoglycans was too low to register (a) but saw a marked improvement in the post-treatment analysis (c). The spectral graphs of each disc level in baseline (b) and post-treatment (d) are shown, there is no graph data for the L1/2 level. Proteoglycan resonance is represented at 2 ppm, and below that, are the acids, predominantly lactate. Note the increased peak of proteoglycans in the post-treatment analysis (d) compared to the baseline analysis (b). SI score: structural integrity score

MRS was performed with the MRI to analyze the lumbar IVD structural integrity and biochemical composition. MRS was utilized as an exploratory tool to analyze possible IVD changes with conservative care. Utilizing NOCISCAN (Broomfield, CO: Aclarion Inc.) post-processing, structural and biochemical markers were charted (Figure 2, panels a, b and Figure 3, panels a, b). The results indicated that the L3/4 and L5/S1 IVDs were the probable pain generators, with significant loss of structural integrity in the L5/S1 IVD.

These quantitative findings confirmed a DLBP diagnosis. Care continued with emphasis on patient reassurance and 16 treatments of Cox flexion-distraction SMT over 16 weeks. There were subjective improvements in pain and functional levels with a full return to weight lifting and golf. However, the patient experienced another exacerbation while getting dressed in the 16th week of care. He rated the pain 6 on the NPRS and the ODI increased to 46%. Based on patient preference, high-velocity low-amplitude SMT was utilized for two treatments over one week. At this time a repeat MRI was utilized to evaluate any qualitative IVD changes (Figure 1, panels c, e). A post-treatment quantitative MRS performed along with the MRI demonstrated marked improvement in the structural and biochemical findings at the L2/3 and L3/4 levels (Figure 2, panels c, d and Figure 3, panels c, d). The patient's symptoms have since resolved, with occasional flare-ups lasting only a couple of weeks with continued treatment.

## Discussion

Qualitative imaging features of IVD degeneration are seen in 40% of people under 30 years of age and in 90% of people over 50 years of age [17]. Many people do not experience symptoms which indicates that IVD degeneration is a common age-related change [9]. However, the severity of IVD degeneration predicts first-time LBP episodes [23]. The structural and biochemical mechanisms of DLBP attempt to explain the transformation from asymptomatic IVD degeneration to symptomatic IVD degeneration, or active discopathy [7,9,19,20].

The NP of healthy IVD resists compression, a function derived from high water and proteoglycan content paired with intact annulus fibrosis (AF) [24,25,29,30]. An early characteristic of IVD degeneration is proteoglycan breakdown. Proteoglycan notochordal cell derivatives decline in quantity with increasing age [31,32]. Because notochordal cells are less abundant, intact proteoglycan content decreases along with reduced imbibition of water and nutrients into the NP. As the NP water content decreases, the ability of the AF to absorb hoop stress forces is reduced, which allows for structural causes of DLBP, such as annular tears and disc herniations [30,33,34]. In disc herniation, the clinical presentation often includes radicular symptoms and, less frequently, centralized pain. In this case, the patient had a disc protrusion but no radicular symptoms further suggesting the pain source was a degenerative IVD and not radicular.

Other considerations in DLBP are biochemical changes. The healthy IVD is avascular, requiring the use of glycolysis as its energy system, which creates lactate as a byproduct. In a healthy IVD with imbibition of water and nutrients in exchange for waste products, lactate levels stay low. Decreased disc imbibition occurs with reduced intact proteoglycan. As the degenerative process continues, cartilaginous endplates calcify further reducing imbibition [32,33]. Lactate accumulation results in reduced intradiscal pH, accelerated degeneration of proteoglycans, hypoxia, and cell death [4,17,21,24]. With increased lactate, there are also increased proinflammatory cytokines, more so in IVDs with adjacent Modic type I changes [7,17,22,32,34].

Structural changes occur synchronously with biochemical changes. A healthy IVD is only innervated in the outer layers of the AF. When annular fissures occur in a degenerated IVD, inflammatory cells and lactate can reach the normally innervated outer layers of the AF. These neural tissues have acid-sensing ion channels making them sensitive to pH changes [4,33-35]. More importantly, biochemical changes and annular fissures create an environment conducive to vascular granulation tissue formation and neoneural growth. New nerves grow into previously aneural areas of the AF and as deep as the NP, where intradiscal pH is low and proinflammatory cytokines are high [17,23,33,34].

While the structural and biochemical causes of DLBP are known, there are no adequate sensitive and specific clinical tests for DLBP. The goal of clinical evaluation of LBP patients should be to identify or rule out serious pathology. Once accomplished, more benign pathoanatomic diagnoses for LBP can be considered. Precise clinical characteristics of DLBP are not well defined and complex pathomechanisms of LBP frequently make diagnosis uncertain. Although centralized LBP is highly sensitive for DLBP, it is not specific [23]. Further complicating LBP are psychosocial factors that impact prognosis and treatment options. These individual contextual factors and lack of specificity in clinical diagnosis lead to significant variability in the management of DLBP patients [5,13].

MRS concept description

A solution to the lack of specificity in the diagnosis of DLBP may be a more specific imaging modality. MRS is a non-invasive method that provides objective quantification of chemical differences in tissues and is used to evaluate specific biomarkers. MRS utilizes the same underlying concept as MRI, where a main magnetic field interacts with protons and radiofrequency currents, but it takes advantage of the fact that individual metabolites have distinct surrounding electron distributions or chemical bonding, which interact slightly differently with the main magnetic field [36]. Consequently, each metabolite resonates at a slightly different magnetic resonance frequency, or chemical shift, allowing for unique identification by their emission spectrum [5,36,37]. Specific to DLBP, Keshari et al. showed metabolites like proteoglycans and lactate serve as spectroscopically quantifiable biomarkers in ex vivo IVD specimens [24].

Recently, Gornet et al. utilized MRS to characterize in vivo metabolic features of painful lumbar IVDs using NOCISCAN technology [21]. NOCISCAN utilizes MRS to assess relative biomarkers for IVD degeneration and pain. Imaging technologists mark a region of interest inside the IVD NP on a conventional MRI sequence to represent a voxel of information. NOCISCAN-LS exam (a proprietary protocol) is run and, with a cloud-based post-processing analysis, creates MRS spectra. The x-axis of the spectral graph describes relative peaks of resonance plotted from right to left and the y-axis describes the degree of chemical shift. These spectral signatures are measured with a diagnostic algorithm to generate a NOCISCORE ratio of proteoglycans to lactate, which has been correlated to provocative discography.

Gornet et al. showed that the spectral measurements of proteoglycans and lactate that were used to create the NOCISCORE score showed reliable identification of painful IVDs when compared to provocative discography and had a significant favorable impact on surgical outcomes [21]. Because provocative discography is painful, invasive, and leads to accelerated degeneration in the injected segments, there is debate about the overall usefulness of the modality [5,15,16]. Gornet et al. showed MRS compared to provocative discography without the associated risks [21].

In our case, the painful IVDs identified by MRS were at L5/S1 which demonstrated qualitative imaging changes, but also at L3/4 which interestingly did not show any qualitative imaging changes. This might suggest that MRS and NOCISCAN technology can identify provocative degenerative IVD changes earlier than qualitative imaging modalities. After treatment, all IVD levels except L5/S1 demonstrated a decrease in NOCISCORE, indicating biochemical improvements. Unfortunately, this patient experienced an exacerbation of pain days prior to the second scan, thus demonstrating an increase in the NOCISCORE at L5/S1. However, the NOCISCORE can also be used to interpret the ratio of the proteoglycans to lactate, where a significant increase in proteoglycans is associated with improved structural integrity, the SI score, as seen at L5/S1 (Figure 2, panels a, c and Figure 3, panels a, c). Alternatively, it may be explained by variability in the temporal expression of molecular scale events from those at tissue levels. Additionally, there is a known disconnect between degenerative structure and function that varies in both frequency and duration. More research could assess IVD structural integrity and pain biomarkers to understand when these changes occur and how they evolve during treatment.

Non-surgical treatments are most commonly utilized in DLBP patients. The most non-invasive option is patient education, which should be employed alongside every other treatment used in accordance with the biopsychosocial patient care model. Patient education includes advice to stay active, assurance of the benignity of DLBP, and its favorable prognosis, which was utilized in our case. Relaxation techniques and cognitive behavioral therapy (CBT) can modify negative thoughts and ruminations about pain/disability and can address any coexisting catastrophization and kinesiophobia [11,28,38].

Other non-surgical treatments include opioid analgesic therapy (OAT), exercise, and SMT. Whedon et al. compared long-term outcomes for patients who initiated LBP care with SMT to patients who initiated care with OAT. They concluded that the SMT group had lower rates of escalated care, thereby reducing overall cost burden [29]. A systematic review and meta-analysis performed by Paige et al. showed statistically significant improvement of SMT similar to NSAIDs, but with only transient minor musculoskeletal harms, whereas nonsteroidal anti-inflammatory drugs (NSAIDs) can be very harmful to certain patient populations [11,39].

SMT is used to treat mechanical aspects of pain, specifically in areas of hypomobility [40]. With a decreased water content in degenerative IVDs, it is theorized that motion segments become unstable or hypermobile. However, there is much literature supporting the idea that the IVD may become stiffer due to the changes in AF collagen [34,41]. This has significant implications for treatment. Improving motion segment mobility may provide better outcomes than surgical fusion in the properly selected patient. In the presented case, Cox flexion-distraction SMT was utilized. Flexion-distraction SMT employs slow manual traction and mobilization of spinal motion segments [42]. The outcomes of Cox flexion-distraction SMT include increased mobility and a decrease in perceived pain. Choi et al. evaluated the biomechanical aspect of flexion-distraction SMT on the IVD and concluded that increased imbibition facilitated the movement of metabolites into the IVD, and increased IVD height [40].

In an appropriate patient, surgical treatment of specific lumbar segments can be beneficial. Gornet et al. showed a significant impact on surgical outcomes in DLBP cases when the correct pathoanatomic target was identified. In their study, Gornet et al. used MRS and NOCISCAN technology to identify painful IVDs. The outcomes were better in the group that received surgical treatment of only the specific levels identified as painful by NOCISCAN compared to the group whose surgical treatment did not include painful IVDs identified by NOCISCAN [21]. The lack of specificity of the correct pathoanatomic target may explain why outcomes of surgical intervention for DLBP have a low success rate.

In this case, post-treatment MRS and NOCISCORE results showed an improvement in structural integrity and biochemical changes. These results imply that conservative management may improve those factors of IVD degeneration and DLBP. The mechanisms to explain why SMT may improve DLBP are variable. It may be as simple as increasing motion at those segments and improving imbibition to restore IVD homeostasis. Another area of study, mechanobiology, proposes that mechanotransduction is the cellular response to mechanical load.

Mechanotransduction is the process cells use to detect and respond to mechanical signals and may be the mechanism underlying the clinical benefits of SMT [43]. There is no evidence to support the spontaneous regeneration of the IVD. However, the notochordal cells within the NP from which proteoglycans are derived, have been shown to differentiate into mature NP cells under mechanical stimulation in animal models [31,33]. Ex vivo studies have demonstrated that both NP cells and AF cells have a strong direct response to external mechanical stimuli. The response is dose-dependent with low magnitude and moderate frequency tensile load promoting proteoglycan production. In contrast to therapeutic effects, elevated magnitude and frequency tensile or compressive loads are catabolic, decreasing proteoglycan production and activating metalloproteinases [4,31,41,43]. Some in vitro studies have also demonstrated that excessive compressive load can upregulate Piezo1 mechanosensitive ion channels, causing elevation of pro-inflammatory cytokines, mitochondrial dysfunction triggering the cellular apoptosis pathway, and reduction of collagen II increasing NP stiffness [44]. More research in this field may help develop appropriate load doses in SMT management of DLBP.

The results of this case report cannot be generalized to a larger population; however, this study further supports that MRS and NOCISCAN technology can identify painful IVDs across a larger sample of patients with DLBP [21]. As this is the first case where post-treatment MRS was performed in a conservatively treated patient, a larger randomized control trial is needed to validate the results of SMT benefit to IVD integrity and to eliminate potential confounding factors.

## Conclusions

We present the utilization of MRS in a conservatively managed patient to confirm the IVD as a pathoanatomic pain source. Baseline and post-treatment IVD structural integrity and biochemical pain profile were quantified. The findings of this case imply that SMT in DLBP management may improve the IVD structural integrity and diminish pain biochemistry. The structural and biochemical contributors to DLBP are complex with many potential confounding factors and more research may provide more understanding.
